# Addressing cancer care inequities in sub-Saharan Africa: current challenges and proposed solutions

**DOI:** 10.1186/s12939-023-01962-y

**Published:** 2023-09-11

**Authors:** Olabode Omotoso, John Oluwafemi Teibo, Festus Adebayo Atiba, Tolulope Oladimeji, Oluwatomiwa Kehinde Paimo, Farid S. Ataya, Gaber El-Saber Batiha, Athanasios Alexiou

**Affiliations:** 1 Department of Biochemistry, University of Ibadan, Ibadan, Nigeria; 2 Department of Biochemistry and Immunology, Ribeirão Preto Medical School, University of São Paulo, Ribeirão Preto, São Paulo, Brazil; 3 Department of Zoology, University of Ibadan, Ibadan, Nigeria; 4 Department of Biochemistry, College of Biosciences, Federal University of Agriculture, Abeokuta, Nigeria; 5 Department of Biochemistry, College of Science, King Saud University, P.O. Box 2455, 11451 Riyadh, Saudi Arabia; 6 Department of Pharmacology and Therapeutics, Faculty of Veterinary Medicine, Damanhour University, AlBeheira 22511 Damanhour, Egypt; 7 Department of Science and Engineering, Novel Global Community Educational Foundation, NSW 2770 Hebersham, Australia; 8 AFNP Med, 1030 Wien, Austria

**Keywords:** Cancer care, Africa, Disease burden, Equity, Public health

## Abstract

**Introduction:**

Cancer is a significant public health challenge globally, with nearly 2000 lives lost daily in Africa alone. Without adequate measures, mortality rates are likely to increase. The major challenge for cancer care in Africa is equity and prioritization, as cancer is not receiving adequate attention from policy-makers and strategic stakeholders in the healthcare space. This neglect is affecting the three primary tiers of cancer care: prevention, diagnosis, and treatment/management. To promote cancer care equity, addressing issues of equity and prioritization is crucial to ensure that everyone has an equal chance at cancer prevention, early detection, and appropriate care and follow-up treatment.

**Methodology:**

Using available literature, we provide an overview of the current state of cancer care in Africa and recommendations to close the gap.

**Results:**

We highlight several factors that contribute to cancer care inequity in Africa, including inadequate funding for cancer research, poor cancer education or awareness, inadequate screening or diagnostic facilities, lack of a well-organized and effective cancer registry system and access to care, shortage of specialized medical staff, high costs for screening, vaccination, and treatment, lack of technical capacity, poor vaccination response, and/or late presentation of patients for cancer screening. We also provide recommendations to address some of these obstacles to achieving cancer care equity. Our recommendations are divided into national-level initiatives and capacity-based initiatives, including cancer health promotion and awareness by healthcare professionals during every hospital visit, encouraging screening and vaccine uptake, ensuring operational regional and national cancer registries, improving healthcare budgeting for staff, equipment, and facilities, building expertise through specialty training, funding for cancer research, providing insurance coverage for cancer care, and implementing mobile health technology for telemedicine diagnosis.

**Conclusion:**

Addressing challenges to cancer equity holistically would improve the likelihood of longer survival for cancer patients, lower the risk factors for groups that are already at risk, and ensure equitable access to cancer care on the continent. This study identifies the existing stance that African nations have on equity in cancer care, outlines the current constraints, and provides suggestions that could make the biggest difference in attaining equity in cancer care.

**Supplementary Information:**

The online version contains supplementary material available at 10.1186/s12939-023-01962-y.

## Introduction

Cancer is a significant global public health challenge, accounting for a substantial number of deaths worldwide. In Africa alone, there were 1,109,209 new cancer cases and 711,429 cancer-related deaths reported in 2020, with approximately 1,949 lives lost to cancer each day [1].

It is projected that there will be a 70% increase in new cancer cases by 2030 due to factors such as population growth, ageing, exposure to carcinogenic agents, unhealthy lifestyles, and tobacco and alcohol use [2–4]. Contributing to the high morbidity and mortality rates are issues such as low awareness, limited knowledge, and unfavourable attitudes towards cancer screening and vaccination, late presentation for cancer diagnosis, high costs of cancer care, and misdiagnosis [2, 5].

Despite the alarming mortality rate, cancer has not received sufficient attention from policymakers and stakeholders in addressing its impact on individuals, families and communities [2]. Cancer has caused pain, deaths, and impoverished families (due to the financial costs of treatments) and we should not delay taking decisive steps in putting an abrupt end to its onslaught on humans. Early detection of cancer significantly improves treatment outcomes, but unfortunately, in low- and middle-income countries, most cancer cases go unreported, and many are diagnosed at advanced stages when the disease has become aggressive or metastasized. Low- and middle-income countries bear a disproportionate burden of the global cancer burden, accounting for approximately 400% and 168% respectively [6].

Cancer care equity is essential in ensuring that everyone, regardless of their socioeconomic status or geographic location, has an equal opportunity to prevent cancer, detect it early, and receive appropriate care and follow-up after treatment. However, achieving cancer care equity faces numerous barriers. These include inadequate funding for cancer research, limited cancer education and awareness efforts, insufficient screening and diagnostic facilities, lack of organized and effective cancer registry systems and access to care, shortage of specialized healthcare personnel, high costs of screening, vaccination, and treatment, inadequate technical capacity, poor response to vaccination, late presentation for cancer screening, and the impact of myths, misinformation, stigma, and socio-cultural/religious beliefs on cancer education and awareness [7, 8]. Addressing these challenges and promoting cancer care equity is crucial in reducing the global burden of cancer and improving outcomes for individuals and communities affected by this disease. Media (including social and print) and healthcare providers have been identified as major sources of cancer information and leveraging these channels can help promote cancer education and awareness [5].

Diagnosis is indeed a significant challenge in cancer care, and many cancer patients in Africa may have to visit multiple healthcare providers before receiving a definitive diagnosis, leading to delays in early diagnosis and presentation of cancer at advanced stages [8]. This issue is compounded by the fact that in low-resource settings, such as sub-Saharan Africa, where the healthcare sector is already burdened with addressing infectious diseases like malaria, HIV/AIDS, acute respiratory infections, and tuberculosis, limited attention is given to non-communicable diseases like cancer [2, 9]. However, government agencies and non-governmental organizations across several countries on the continent have been putting in measures to address this menace, especially in terms of making provision for opportunistic or community-based cancer screening for breast, cervical, prostate, and testicular cancer.

Another major challenge in achieving cancer care equity in Africa is the high cost of cancer screening, vaccination, and treatment. Most costs are paid out-of-pocket, and with a significant proportion of the population living in extreme poverty (33% in sub-Saharan Africa), many individuals cannot afford these services, leading to abandoned therapies, low response to screening, and poor vaccination rates [10]. To save as many African lives as possible from the impact of cancer, it is crucial to prioritize the three main tiers of cancer care: prevention, diagnosis, and management or treatment.

Nationwide and sector-based initiatives are important in addressing the challenges to cancer care equity in Africa. These initiatives could include cancer health promotion and awareness, where healthcare providers utilize every opportunity during hospital visits to educate patients about cancer diagnosis and screening. Other measures could include establishing functional national and regional cancer registries, improving screening and vaccine uptake, enhancing budget allocation for the healthcare sector personnel, facilities, and capacity building or specialty training, increasing funding for cancer research, expanding insurance coverage for cancer care, and utilizing telemedicine and mobile health platforms for remote diagnosis [10].

The theme for World Cancer Day 2023 was “Close the Care Gap,” emphasizing the global focus on achieving equitable access to cancer care. This study aims to identify the status of African countries in terms of cancer care equity, explore the existing limitations, and provide recommendations that could contribute to achieving equitable access to cancer care in Africa. By addressing these challenges and implementing targeted interventions, we can work towards reducing the burden of cancer in Africa and improving outcomes for individuals and communities affected by this disease.

## Materials and methods

### Search strategy

A systematic literature search was conducted between November 2022 and March 2023 using Google Scholar and PubMed databases. The search utilized the following keywords: “cancer care,” “cancer burden/incidence,” and “cancer care equity/inequity.” To focus specifically on the context of sub-Saharan Africa, the search was restricted to countries within the region, reflecting a continental review while corroborating this with other earlier studies describing efforts in other regions. Preprints without peer-review, review articles, and studies that did not align with the objectives of this review were excluded. Inclusion criteria were article that met the study objectives focused on cancer care equity/inequity in sub-Saharan Africa, ongoing efforts to address these challenges and current standpoints. Search results were likewise filtered based on title, abstract, and availability of free full text. The following Authors; OEO, and FAA, TOO and JOT critically evaluated the search results on PubMed and Google Scholar respectively, to come up with a final list of articles that are well suited and used for this study. In addition to academic sources, reputable web links such as the International Agency for Research on Cancer (IARC), World Health Organization (WHO), World Poverty Clock, The Cancer Atlas, and the National Cancer Institute were consulted to gather further information relevant to the study’s objectives. This comprehensive search strategy aims to obtain a thorough understanding of the topic of addressing cancer care inequities in sub-Saharan Africa.

### Cancer risk factors

Cancer is a multifactorial disease influenced by a complex interplay of genetic, environmental, and lifestyle factors [7, 11]. While some risk factors, such as genetics, cannot be modified, many lifestyle factors, including tobacco and alcohol use, diet, physical activity, and weight management, can be modified to reduce the risk of developing cancer. Genetic mutations or inherited gene mutations account for approximately 5–10% of all cancers, with some cancers having a stronger genetic component, such as breast and ovarian cancer in individuals with BRCA1 and BRCA2 mutations [7]. Environmental factors are estimated to cause about 20% of all cancers worldwide, with exposure to carcinogens such as UV radiation, ionizing radiation, and chemical carcinogens (e.g., asbestos, benzene) playing a significant role [11]. Infections from bacteria and viruses also contribute to cancer risk, with human papillomavirus (HPV) being a known risk factor for cervical cancer, and Hepatitis B and C infections increasing the risk of liver cancer [11].

Lifestyle factors also play a crucial role in cancer risk. Tobacco use is a well-established risk factor for various types of cancer, accounting for nearly one-third of all cancer deaths globally, and is the leading preventable cause of cancer [7, 11]. Alcohol consumption is another modifiable risk factor, with heavy or prolonged alcohol use increasing the risk of several types of cancer, including breast, liver, and mouth cancer [11]. Diet is also a significant contributor to cancer risk, with a diet high in processed and red meat, low in fruits and vegetables, and lacking in dietary fiber increasing the risk of colorectal cancer [8, 11]. Physical inactivity and obesity are also important risk factors, with obesity being associated with an increased risk of developing several types of cancer, including breast, colorectal, endometrial, kidney, and liver cancer [9–11].

Globally, approximately half of cancer-related deaths are attributed to modifiable risk factors, including sedentary lifestyle, obesity, and uncontrolled alcohol and tobacco use [12]. In low- and middle-income countries with less enforcement of labour and environmental protection laws, occupational exposure to carcinogens is a significant concern [12]. Occupational exposure to carcinogens, such as radiation, benzene, soot, asbestos, arsenic, nickel compounds, formaldehyde, etc., has been found responsible for 3–6% of the global cancer burden [12]. Tobacco use has been implicated in about 22% of cancer-related deaths, particularly in developed and high-income countries [3]. Infections, such as human papillomavirus (HPV) and hepatitis B and C, have been linked to approximately 25% of cancer-related cases in low- and middle-income countries [3].

For example, in some parts of Africa, increased and indiscriminate use of tobacco, cannabis, and alcohol has been linked to an increased incidence of lung cancer [2, 13]. Furthermore, the adoption of Western lifestyle factors, such as nulliparity, nutrition, use of oral contraceptives, and post-menopausal hormone replacement therapy, has contributed to a dramatic increase in breast cancer cases in Southern and Northern Africa from 23.3 cases per 100,000 people in 2002 to 48.9 cases per 100,000 people in 2018. In contrast, there was no significant increase in Western, Eastern, and Central Africa, which may be related to reduced urbanization and lower exposure to environmental carcinogens [2].

Reducing exposure to modifiable risk factors, such as tobacco and alcohol use, improving occupational safety measures, and promoting healthy lifestyle behaviours, can significantly lower an individual’s risk of developing certain types of cancer. Non-modifiable factors, such as aging, gender, family history, inherited genetic mutations, and race/ethnicity, have been identified as significant contributors to cancer risk. While individuals have little or no control over these factors, understanding their impact can help in taking proactive steps to reduce modifiable risk factors, such as avoiding exposure to carcinogens, getting vaccinated for preventable infections, and participating in routine screenings for early detection and management of cancer.

Aging is a major factor in cancer incidence and mortality. While cancer can be diagnosed at any age, most cases are reported between 36 to 55 years, and the risk of developing most types of cancer increases with age. Gender also plays a role in cancer risk. Breast and cervical cancers are more prevalent in women, while prostate and testicular cancers are more frequent in men. African American men have been found to have 2.5 times the risk of dying from prostate cancer compared to European American men. However, due to possible side effects of therapies, such as sexual dysfunction and incontinence, men of African ancestry may be less likely to seek care compared to European Americans [2].

Family history and inherited genetic mutations also contribute to cancer risk. Individuals with a family history of cancer or those who carry inherited genetic mutations associated with cancer, such as BRCA1 and BRCA2 mutations, may have an increased risk of developing certain types of cancer. Race and ethnicity also play a role in cancer risk. Certain racial and ethnic groups may have higher or lower risks of developing specific types of cancer. For example, African Americans have been found to have higher risks of developing cancers such as prostate, breast, and colorectal cancers, while Asians may have lower risks of developing certain types of cancer. Despite the non-modifiable nature of these risk factors, it is important to be aware of them and take steps to reduce modifiable risk factors, such as avoiding exposure to carcinogens, getting vaccinated for preventable infections, and participating in routine screenings for early detection and management of cancer [2].

### Cancer statistics – incidence and mortality

The global burden of cancer is significant, with an estimated 19,292,789 new cancer cases reported in 2020 (Table 1). Asia had the highest incidence rate, accounting for 9,503,710 (49.3%) of the cases, followed by Europe with 4,398,443 (22.8%), North America with 2,556,862 (13.3%), Latin America and the Caribbean with 1,470,274 (7.6%), Africa with 1,109,209 (5.7%), and Oceania with 254,291 (1.3%) [14]. These statistics highlight the global burden of cancer and the variation in cancer incidence across different regions. It is important to understand the trends in cancer incidence and mortality to inform cancer prevention and control strategies, including efforts to reduce modifiable risk factors, promote early detection and screening, and improve access to cancer care and treatment worldwide.

**Table 1:** Inter-continental comparison of estimated new cancer cases, risk and deaths

Continent	Population	New cases	Risk (%)	Top New cases	Number of deaths	Risk (%)	Top death cases
**Africa**	1,340,598,088	1,109,209	13.7	Breast Cervical Prostate	711,429	9.3	Breast Cervical Liver
**Latin America and the Caribbean**	653,962,327	1,470,274	18.9	Prostate Breast Lung	713,414	9.0	Lung Breast Prostate
**North America**	368,869,643	2,556,862	33.9	Breast Lung Prostate	699,274	9.2	Lung Pancreas Breast
**Asia**	4,639,847,464	9,503,710	17.5	Lung Breast Stomach	5,809,431	10.9	Lung Liver Stomach
**Europe**	748,843,410	4,398,443	28.2	Breast Lung Prostate	1,955,231	11.7	Lung Colon Breast
**Oceania**	42,677,809	254,291	37.5	Breast Prostate Skin melanoma	69,354	9.3	Lung Colon Breast

From Table 1, breast cancer is estimated to be one of the top three most frequent cancers in terms of incidence and mortality in all continents, except for Asia where it is the fifth most frequent cause of cancer death. Oceanians (37.5%) and North Americans (33.9%) have the highest risk of developing cancer before the age of 75, while Europeans and Asians have the highest risk of dying due to cancer-related deaths before the age of 75 [1]. However, Africans have a relatively fair chance of cancer risk, with both incidence and mortality rates being comparatively lower than other continents.

The availability of data on cancer incidence and mortality rates in Africa is limited. Incidence rates were obtained from country-specific data sources in only 30 countries (55.6%), while local registries provided data for 8 countries (14.8%). Additionally, 16 countries (29.6%) had no data, so estimates were derived from data from neighbouring countries [14]. Similarly, only a few African countries had national incidence estimates for cancer-related deaths, with most countries relying on data from neighbouring countries (46, 85.2%). This suggests that many cancer-related deaths in Africa may go unreported or are misdiagnosed, raising questions about the functionality of national cancer registries in the region. As a result, real-time surveillance and data-driven policies for cancer care may be lacking in many African countries, highlighting the need for improved data collection and evaluation of existing cancer care policies (Supplementary File 1).

### Cancer statistics – screening and vaccination

Cervical cancer has been reported as one of the top three most frequent cancers in Africa (Table 1), in contrast to its low prevalence in developed and high-income countries [9]. Earlier reports have also identified cervical cancer as one of the most frequent cancers in sub-Saharan Africa [4, 7]. In fact, in 2018, 19 out of the 20 countries with the highest cervical cancer burden were located in sub-Saharan Africa [15], highlighting the significant burden of this preventable cancer in the region.

Cervical cancer is highly preventable and has a high cure rate when detected early [3]. However, poor access to screening, vaccination, and treatment has contributed to about 90% of cervical cancer-related deaths [15]. A previous study involving 1,006 participants from Nigeria and Egypt found that only 11.7% of participants were aware of HPV vaccines, 11% had undergone screening, and a mere 2.2% had received HPV vaccines [5]. The World Health Organization’s (WHO) HPV national vaccination program has been helpful in many Southern and Eastern African countries [2]. However, more efforts are needed to ensure equitable access to HPV vaccines by reducing costs or including them in national vaccination schedules, which can help in flattening the curve of cervical cancer burden in Africa.

Promoting opportunistic and carefully designed awareness, vaccine, and screening uptake programs will play a significant role in reducing the burden of cervical cancer in Africa. These efforts may include public health campaigns to increase awareness about cervical cancer prevention and the importance of HPV vaccination, as well as initiatives to improve access to screening services, particularly in underserved areas. Additionally, collaboration among policymakers, healthcare providers, and communities can help to develop and implement effective cervical cancer prevention and control programs that are tailored to the unique needs of African populations. By addressing barriers to screening, vaccination, and treatment, and promoting comprehensive cervical cancer prevention strategies, it is possible to make progress in reducing the burden of cervical cancer in Africa and improving overall health outcomes for women in the region.

### Limitations to cancer care

Several limitations hinder adequate cancer care in Africa, including lack of awareness, high cost of treatment and research funding, expertise shortage, and shortage of healthcare workers, such as oncologists and specialized cancer care nurse. These limitations have resulted in a disproportionate burden of cancer and high mortality rates in most African countries, particularly in sub-Saharan Africa (SSA). For instance, countries in the WHO African region had an estimated 811,200 new cancer cases (4.5% of the world population) and 534,000 cancer deaths (7.3% of the total world) in 2018 [16]. Moreover, 70% of newly diagnosed cancer cases occur in Africa, where the survival rate of cancer is “30% to 50% lower than that of high-income countries” [17].

In comparison to other continents, limitations in cancer care have led to reduced survival rates for cancer patients in Africa [18]. For instance, the 5-year survival rate for women with breast cancer is 82% in Europe, while it is 46% in Uganda, less than 39% in Algeria, and 12% in the Gambia [19]. These limitations also impose significant socio-economic burdens on the affected population, as governments and families in these countries struggle with the increasing cases of cancer on the continent. For example, a case study conducted in Kenya found that households of cancer patients incurred significant economic losses due to lost wages, sold assets, and debts from medical (drugs and diagnostics), as well as non-medical spending (such as transportation and accommodation) on cancer care, despite being enrolled in the National Hospital Insurance Fund (NHIF) which covered treatments such as radiotherapies, chemotherapies, and surgeries [20].

The prospects of overcoming the cancer burden in Africa appear bleak unless these limitations are addressed. According to forecasts, countries in the African region are projected to have a cancer burden greater than the rest of the world between 2020 and 2040 [21]. Specifically, Sharma et al. [22] project that “nearly 2.1 million cases and 1.4 million deaths are projected by 2040 in Africa; this, however, can be even higher due to escalation of behavioural and environmental risk factors, increasing life spans, and improvements in cancer registration.” Therefore, an urgent and rational national cancer control planning is needed in sub-Saharan Africa (SSA) to overcome these limitations [14].

### Misinformation and myths

Misinformation and myths about cancer can greatly hinder cancer screening and vaccination efforts in Africa. These misconceptions include beliefs such as cancer being a disease only prevalent in developed nations, wealthy or aged individuals, or being a punishment for wrongdoing or a generational curse. These myths can lead to fear, stigma, and avoidance of seeking medical care, which can delay diagnosis and treatment and negatively impact cancer outcomes [23].

Addressing these misconceptions and misinformation is crucial to improving cancer care in Africa. Communication channels such as social media, press, TV, and radio can be utilized to disseminate accurate information about cancer. Religious and traditional leaders, who hold significant influence in many African communities, can also be engaged to help educate their communities or followers about cancer and dispel myths. Education campaigns that provide evidence-based information about cancer, its risk factors, prevention, and early detection can help raise awareness and increase uptake of cancer screening and vaccination programs [5, 23].

Efforts should also be made to engage local communities and tailor cancer education messages to local cultural and societal norms. This can help in building trust and addressing specific beliefs or concerns related to cancer. Collaborations between healthcare providers, community leaders, and other stakeholders can play a crucial role in disseminating accurate information about cancer and addressing misconceptions [5]. Comprehensive and culturally sensitive education campaigns, involving various stakeholders and leveraging different communication channels, can help raise awareness, dispel myths, and promote early detection and appropriate medical care for cancer patients.

### Costs of treatment

The cost of cancer care is a significant challenge, both in high-income countries like the United States and in low-income countries like those in Africa. In the United States, the National Cancer Institute estimates that the average cost of medical care and drugs for cancer treatment can exceed $42,000 in the year following a cancer diagnosis, with some treatments costing up to or exceeding $1 million [24, 25]. These high costs can place a tremendous financial burden on cancer patients and their families.

In Africa, where average monthly incomes are much lower compared to high-income countries, the affordability of cancer care becomes even more challenging. The average monthly income for working groups in Africa in 2022 is around $758, which is about ten times lower than typical incomes in the United States and the United Kingdom [26]. This means that cancer treatment costs can amount to nearly five times the average yearly earnings of a worker in Africa, making it extremely difficult for patients to afford the necessary medical care.

Data on patient spending on cancer care in Africa is scarce, but it is clear that the economic burden of cancer care is significant. Governments in Africa spend much less on health costs compared to high-income countries, with less than $100 per capita, compared to about $8,000 in the United States [21]. This further exacerbates the challenge of accessing affordable cancer care for patients in Africa. The high costs of cancer care can lead to financial strain, debt, and even bankruptcy for cancer patients and their families, and may result in delays or discontinuation of treatment due to inability to afford the costs. This can have a detrimental impact on cancer outcomes and survival rates.

Efforts are needed to address the economic burden of cancer care in Africa, including advocating for affordable and accessible cancer treatments, increasing investment in healthcare infrastructure, and exploring innovative financing models. This may include government-funded health insurance programs, cost-sharing initiatives, and partnerships between governments, NGOs, and other stakeholders to make cancer care more affordable and accessible for patients in Africa. Additionally, efforts to raise awareness about the importance of health insurance, financial planning, and support services for cancer patients and their families can also help mitigate the financial burden associated with cancer care [27, 28].

### Research and funding

**Fig. 1:**
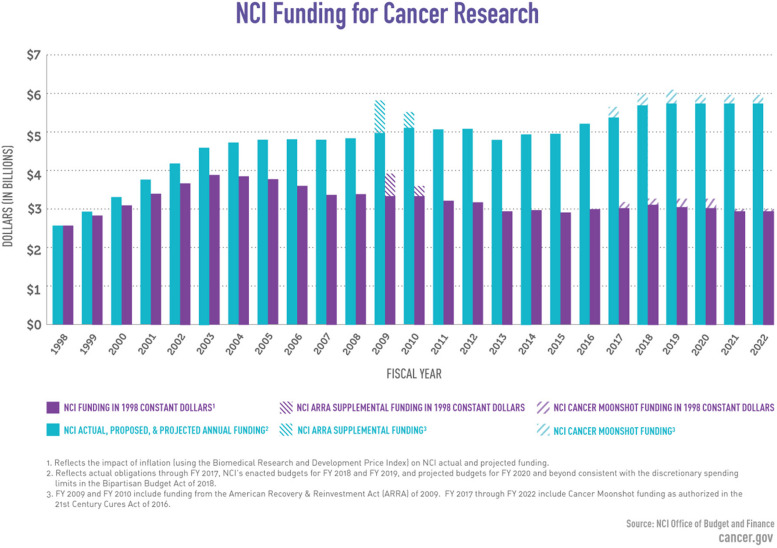
NCI funding for cancer research in the USA

Research on diseases, including cancer, often receives low priority in Africa, and funding for disease research is limited, low, or unavailable in some cases [2, 29, 30]. This lack of funding has a significant impact on the research outputs of African scientists, making it challenging to develop diagnostics, drugs, and care initiatives for cancer patients in Africa. In contrast, a substantial percentage of research funding and efforts is dedicated to cancer research in the United States and Europe. For example, the American Cancer Society (ACS) has invested more than $5 billion in research grants since 1946, and in 2019 alone, they invested over $145.9 million in cancer research [2]. The National Cancer Institute (NCI) in the year 2022 has a total of $6.4 billion available for spending, with $194 million in funding for the Cancer Moonshot℠ and $50 million for the Childhood Cancer Data Initiative [2] (Fig. 1).

Funding is the major limitation to healthcare for diseases including cancer in Africa. The deficiency of funding has led to setbacks in the treatment of common cancers that affect Africans such as breast, cervical, ovarian, prostate, and lung cancers.

Additionally, the lack of funding has also compounded into less expertise in oncology as scientists lack resources such as library access to journals, molecular biology or diagnostics laboratories, administrative assistance because of low prioritization for cancer research, and training in the methodology of cancer research [21].

The disparity in research funding between Africa and high-income countries like the United States and Europe can impact the development of cancer care initiatives, including access to cutting-edge diagnostics, treatments, and supportive care for cancer patients in Africa. It underscores the need for increased investment in cancer research and healthcare infrastructure in Africa to address the burden of cancer and improve outcomes for patients. Efforts are needed to prioritize and increase funding for cancer research in Africa, including engaging governments, philanthropic organizations, and international stakeholders to support research initiatives. Collaborations between African scientists and researchers from other countries can also help foster knowledge exchange and capacity building in cancer research in Africa. Additionally, advocacy efforts to raise awareness about the importance of cancer research and its impact on cancer care in Africa can also help drive increased funding and support for research efforts in the region [2, 29, 30].

### Poor health systems and lack of data infrastructure

The health system in Africa faces significant challenges, including a lack of uniform data registry infrastructure for cancer, despite improvements in cancer epidemiology strategies [2, 31]. This limitation has led to incomplete characterizations of the geographic distribution and determinants of cancer, exacerbating risk factors, reducing awareness, and hindering patient tracking and referral for preventative care. For instance, in low and middle-income regions like Sub-Saharan Africa (SSA), the lack of awareness about risk factors associated with lung cancer, such as smoking, exposure to asbestos, fumes, dust, and insecticides, has contributed to increased cases, in contrast to upper-income regions where cases of lung cancer have significantly decreased due to increased awareness [2, 32, 33].

Furthermore, population-based cancer registries (PBCR), which are crucial data hubs for cancer research, national and regional planning, and preventative efforts, are scarce in most African countries [34]. In places where they exist, the data may be unreliable due to inadequate surveillance and underdiagnosis of cancer cases [35]. As a result, the burden of cancer in SSA may be higher than reported, as current figures may represent a conservative estimate due to the poor health systems and inadequate cancer registries in SSA [3, 17].

To address these challenges, it is imperative to improve the data infrastructure for cancer in Africa, including the establishment of reliable and comprehensive population-based cancer registries. This would enable a better understanding of the true burden of cancer, identification of risk factors, and implementation of effective preventative measures. Additionally, efforts should be made to raise awareness about cancer risk factors and promote early detection and intervention strategies in low and middle-income regions, such as SSA, to mitigate the increasing burden of cancer in these regions [2, 32, 33].

### Diagnostic infrastructure and expertise

According to the World Health Organization (WHO) [3], cervical cancers in many parts of Africa are often not identified or treated until advanced stages due to insufficient access to reproductive health care services, effective screening, and early treatment. Even when an individual is diagnosed, they may not seek treatment, and those who do seek treatment are not guaranteed better outcomes. The WHO reports that fewer than 30% of patients diagnosed with cancer in low-income countries have access to treatment, compared to 90% in high-income countries. This disparity can be attributed to several factors, including limited access to medical care, high costs of treatment, lack of expertise in surgical and cancer specialists, and limited availability of treatment options such as radiotherapy or chemotherapy. These factors collectively contribute to the challenges faced in addressing cancer in Africa, particularly in low-income countries, and highlight the urgent need for improved access to quality cancer care and treatment options.

**Fig. 2:**
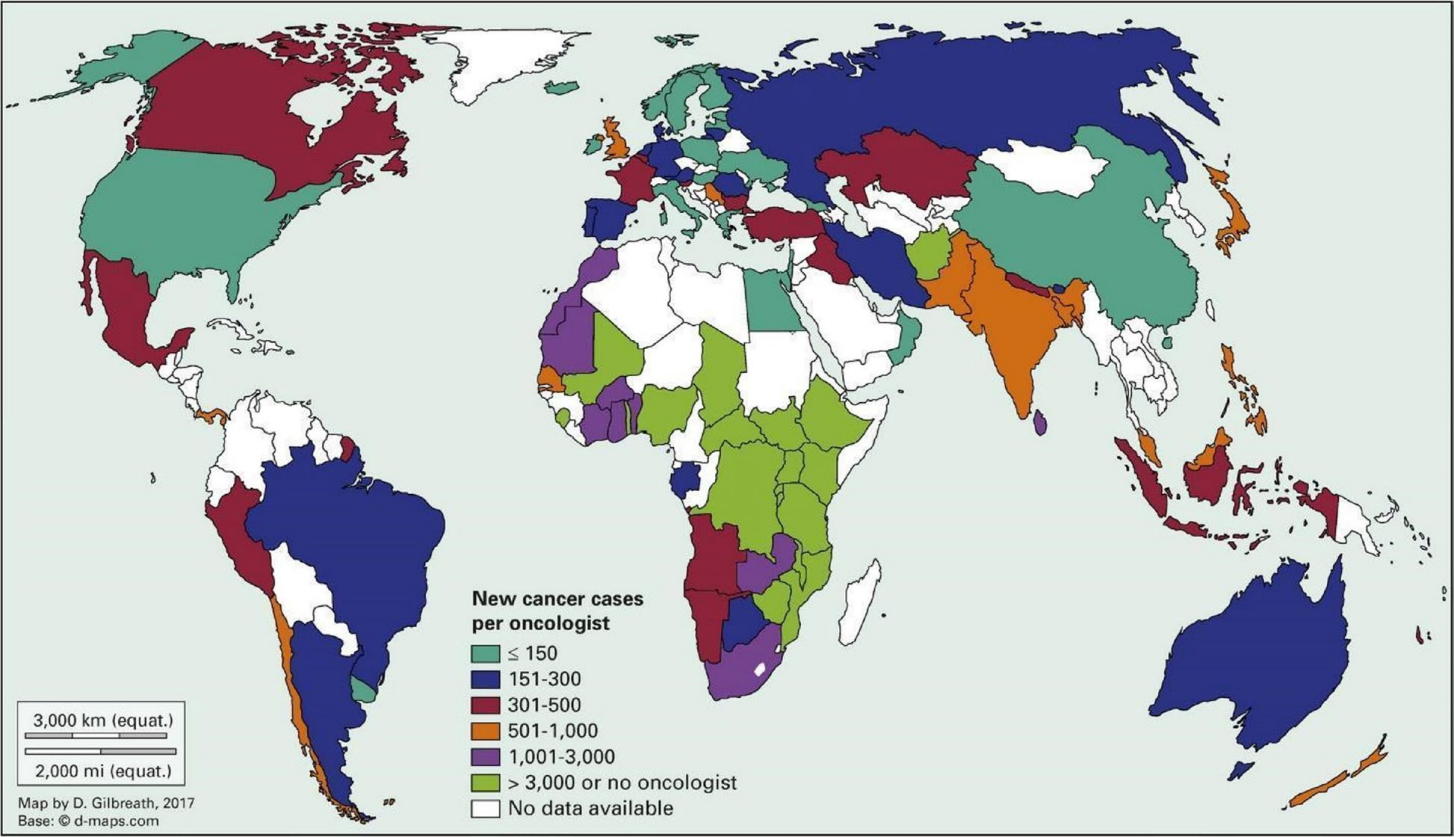
Graphical summary of the availability of oncologists

Africa faces a significant shortfall in the number of healthcare workers, particularly in Sub-Saharan Africa (SSA). In 2006, only about 3% of the world’s healthcare workers lived and worked in SSA [36]. However, this number has declined drastically to 1.3% [37, 38], and it is projected to further decrease by 6.1 million by 2030 as a result of many factors amongst is emigration backed up by globalization [39]. Furthermore, reliable data on the number of oncology specialists in SSA is lacking, but it is assumed to be quite low compared to other continents, given the limited availability of specialized training in cancer treatment [21] (Fig. 2). This shortage of healthcare workers and oncology specialists further compounds the challenges of addressing cancer in Africa, as it results in inadequate capacity for cancer screening, diagnosis, treatment, and follow-up care, contributing to delayed diagnoses and suboptimal management of cancer cases.

Furthermore, studies such as the one conducted by Vanderpuye et al. [40] have highlighted the significant workload faced by African oncologists, with a higher patient-to-oncologist ratio due to shortages of oncology specialists to cater to the large number of cancer patients. For instance, in countries like Cameroon, there are only four medical oncologists and three radiologists serving a population of 26.55 million people [30, 41]. In some African countries, there are no trained oncologists available to treat cancer patients, with countries such as South Sudan, Lesotho, Benin, Gambia, and Sierra Leone reported to have no oncologists [42]. This scarcity of oncologists and other specialized healthcare professionals further exacerbates the challenges of cancer care in Africa, resulting in delayed diagnoses, limited treatment options, and inadequate follow-up care for cancer patients.

Significant progress is being made through training programmes for oncologists in some parts of Africa. For example, in Zimbabwe, there are currently 14 active oncologists for a population of 14.8 million according to its 2020 population estimates, which is a high number compared to some other African countries. However, this ratio of oncologists per population is still low, which is 1 per 1.057 million people when compared to 1 oncologist per 26,418 people in the USA [43].

“In 39 countries (42%), a clinical oncologist would provide care for > 500 patients with cancer, of which 26 countries were in Africa (81%), nine were in Asia (47%), two were in Europe (6%), and two were in South America (29%). An extreme shortage of clinical oncologists— > 1,000 incident cancers per clinical oncologist—existed in 25 countries in Africa (78%) and two countries (11%) in Asia. None of the countries in Europe or North or South America faced such an extreme shortage of clinical oncologists.” (*Image and text adapted from* Matthew [42]).

Because of this expertise shortfall, diagnosis is late with at least 80% of patients in Africa diagnosed with cancer at an advanced stage [44]. Consequently, late diagnosis has increased the mortality burden with the ratio of mortality in Africa (0.66) higher than in high-income countries in Northern Europe (0.40) and the United States (0.29) [1].

The expertise shortfall in Africa has resulted in late diagnosis, with at least 80% of cancer patients being diagnosed at an advanced stage [44]. This late diagnosis has increased the mortality burden in Africa, with the ratio of mortality (0.66) being higher than in high-income countries in Northern Europe (0.40) and the United States (0.29) [1]. Furthermore, a lack of cancer-specific infrastructure and limited access to care and treatment exacerbates this problem. In many cases, patients are unable to access even basic cancer treatments due to the high cost of care and treatment, particularly for those without health insurance or those living in rural areas.

### Corruption and misappropriation of funds

These are significant obstacles to improving healthcare in Africa, including cancer care. Despite receiving substantial international aid, many countries in Sub-Saharan Africa have struggled to make significant progress due to corruption [45]. A US government website reports that 85% of its international donations for health were directed toward Africa, yet the impact of this funding has been limited by corruption [21]. In many cases, funds intended for cancer treatment and research are diverted to other areas or misused, preventing patients from receiving the care they need. This is a significant challenge that must be addressed to improve cancer outcomes in Africa.

### Impact of cancer care inequity in Africa

The impact of cancer care inequity in Africa is significant, with a higher death rate compared to other regions due to inadequate cancer treatment [19, 22, 46–48]. The inequality in cancer care is predicted to lead to a considerable increase in the incidence and mortality rate from cancer in Africa between 2020 and 2040 [22, 47]. In the next 20 years, it is anticipated that the death rate will surpass 30% just in Africa. This is due to disparities in diagnosis, treatment, cancer care facilities, and screening sites [49, 50].

Late presentation of cancer also contributes to increased fatality rates, with breast, prostate, lung, cervical, liver, and esophageal cancers having a higher incidence and death rate, while risks for stomach, thyroid, and bladder cancer are relatively low [47]. Breast cancer is more common in women in Northern and Southern Africa than in other parts of Africa, but it still kills fewer people in those two regions. However, the incidence rate of prostate cancer in men is rising alarmingly, with an increase of more than 60% from 2018 to 2020, and a higher death rate than lung cancer. Cervical cancer is more prevalent in North Africa, and in east, central, and west Africa, more than 75% of infected women die from the disease, with the fatality rate only declining in the south. The fatality rate has risen in all African nations except for the North, where the incidence is very low [51], and an increase in smoking activity is contributing to a difference in aggressive lung cancer with less target therapy, diagnosis, and screening. Stomach cancer has also been reported in Africa with a median overall survival of 9–12 months after metastatic disease has developed [46]. Men are reported to have more deaths than women, and an elevated metastasis and death rate have been linked to colorectal cancer [52].

Cancer survival rates are an important measure of the effectiveness of cancer care in different regions. However, after a cancer diagnosis, the survival rate can vary widely depending on the location [53, 54]. Population-based studies have shown that African countries generally have lower cancer survival rates compared to American and Asian countries [55–57]. For example, Gambia and Uganda have low cancer survival rates, while Turkey, Singapore, and South Korea have high survival rates, and China, Saudi Arabia, Pakistan, and the Philippines have intermediate rates [58]. In countries such as China, South Korea, and Singapore, the survival rates for certain cancers, such as breast, cervical, bladder, and colon cancer, are notably high, ranging from 63–82%. However, in Gambia and Uganda, the only cancer with a survival rate higher than 22% and 13%, respectively, is breast cancer, with a rate of 46%. These differences in survival rates can be attributed to factors such as late detection, delayed presentation, lack of diagnosis, and limited access to cancer screening sites.

Cancer survival rates are an important indicator of the effectiveness of cancer care and can vary widely depending on the level of human development in a given country or region. An earlier study [59] examined survival projections in Central and South America, Asia, and Africa based on their Human Development Index (HDI) and found that survival estimates increased with higher HDI scores [59]. For example, Israel, a country with a high HDI, has a prostate cancer survival rate of 96.8%, while Côte d'Ivoire, a country with a lower HDI, has a 3-year survival rate of 54.6% (with a 95% confidence interval). Additionally, within regions, different cancer types have varying median 3-year survival rates based on their HDI. Lung and stomach cancer have low median survival rates (less than 30%), while cervical and colorectal cancer have high median survival rates (greater than 80%). Breast and prostate cancer have the lowest median survival rates (less than 30%). These disparities can be attributed to the lack of effective cancer healthcare services and the level of development of the nation.

### The WHO effort in addressing cancer care inequity

The World Health Organization (WHO) has recognized cancer as a global public health priority that require urgent attention and has been actively involved in efforts to address cancer care inequity, both in Africa and globally. As part of its mission to promote health for all and achieve the much-desired universal health coverage, there have been ongoing efforts towards reducing the burden of cancer, improving access to cancer care, and ensuring that no one is left behind in the fight against cancer. In Africa, where cancer care challenges are particularly significant due to limited resources and multiple competing health priorities, the WHO has been playing a crucial role in supporting countries in their efforts to tackle cancer care inequity. We provide an overview of the WHO’s initiatives and strategies aimed at addressing cancer care inequity in Africa and globally, and their potential impact in bridging the gap in cancer care. The WHO’s initiatives in Africa and globally are guided by its global cancer strategy, which aims to reduce the global cancer burden, improve cancer prevention and control, and ensure equitable access to timely and quality cancer care.

Such effort includes health promotion and awareness campaigns to raise awareness about cancer risk factors, promote healthy lifestyle choices, and encourage early detection and screening [15]. They have also been advocating for the establishment of functional national and regional cancer registries to improve cancer data collection, analysis, and reporting, which is vital for evidence-based decision-making. Efforts to improve access to cancer screening and vaccination have also been a priority for the WHO in Africa. This includes supporting countries in strengthening their capacity for cancer screening and increasing uptake of cancer vaccines such as the human papillomavirus (HPV) vaccine for cervical cancer prevention, advocating for improved budgeting for the healthcare sector in Africa, including increased funding for cancer care, and exploring innovative financing mechanisms for cancer care [15].

Capacity building, specialty training for healthcare personnel and increased investment in cancer research have been another focus of the WHO in Africa to improve cancer care. This includes efforts to strengthen the skills and expertise of healthcare providers in cancer diagnosis, treatment, and management, as well as promoting interdisciplinary collaboration and coordination among different healthcare providers involved in cancer care. An increased investment in cancer research in Africa would help in the generation of evidence to work with for effective cancer prevention, control, diagnosis, treatment, and management strategies.

### Solutions and recommendations

#### Awareness

The greatest concern with the surging cancer cases in Africa is that they are presented at late stages when the chance of successful treatment is reduced. This ugly trend is largely so because some people are unaware that cancer is real, misinformation or myths about cancer are still being propagated and believed, asymptomatic cases especially at early stages, or due to lack of funds for treatment [5]. Before recent times, many believed that cancer is a disease of the privileged who have access to an ultramodern lifestyle and processed foods and as such not a disease people of low- and middle-income countries should worry about. Now the tables have turned, developed countries have found ways to better manage different cancer types and due to gene-environment interaction and globalization, unheard and uncommon cancer types are beginning to manifest amongst people who never taught they could have cancer, and worse still the health system in most countries in Africa was not prepared for this.

If a disease is claiming the lives of about 1,949 of us daily then we need to talk about it more often on traditional and social media platforms, in our schools, in marketplaces, in public places, in our communities and everywhere. As cancer knowledge and campaign programmes are gaining prominence in sub-Saharan Africa, more people are beginning to realize that the disease is real but how much correct information people have about cancer is what cannot be ascertained. A structured system of disseminating correct information and translating scientific findings into basic knowledge that laymen can understand is crucial hence, the need for more for scientific communicators. In this case, the population needs to hear from the horse’s mouth; the medical practitioners or primary healthcare givers, cancer survivors, the oncology researchers, and all those who are at the forefront of managing cancer. Educational charts in different African languages highlighting different cancer types and common symptoms would help people identify markers and present them for early diagnosis which would in turn reduce mortality from cancer types that can be treated. Structured training on breast self-examination and the importance for women would prove vital in the early diagnosis of breast cancer cases.

For instance, individuals need to know early in their life if they are predisposed to developing cancer so prevention action can start early. Genetic markers for different cancer types should periodically be screened for amongst the population, this would reduce the occurrence of late diagnosis and mortality from this subset. Human genome sequencing is a great tool that can be employed to identify individuals that are predisposed to cancer and other public health diseases, this would equally help the continent to attain genomic data representation globally and in turn, lead to the design of precision medicine for the populace [60]. As promising as this is, there is still a huge gap in terms of advances in genetics and genomics studies in the continent [60].

#### System for early detection

The practice for many people is that a hospital is only an option in a critical situation, as such periodic check-up is not a common practice amongst many Africans. This unhealthy practise contributes significantly to the late diagnosis of cancer. The importance of constant medical check-ups cannot be underestimated for a healthy lifestyle and early detection of diverse life-threatening diseases. For instance, many stillbirths and birth complications have been prevented because pregnant women visit the hospital regularly for a check-up and complications can be detected during those visits. Should we imagine a case where pregnant women decide not to visit the hospital because they do not have labour pain until the occasion of nine (9) months of pregnancy or the due date for delivery, then probably many of us might have died or been born with birth-related complications. Policies should be implemented to encourage opportunistic and periodic check-ups for all age groups and especially for people who are predisposed to developing cancer and other pre-disposing co-morbidities.

Several factors equally deter people from visiting the hospital periodically, some of which are the long waiting period and the insufficient doctor-to-patient ratio in many hospitals. These factors discourage people from visiting the hospital and could even leave them sicker than they came to the hospital. There is a dire need for more hospitals and professionals who can attend to the routine check-up need of individuals without the hassle of long waiting hours. Primary Health Care centres which are the only source of health care in many rural communities also need the capacity to meet the need for early detection.

#### Cancer care through a health insurance scheme

As earlier mentioned, cancer treatment is expensive, and a lot of people affected in Africa cannot afford to pay for treatment at the time of diagnosis. Insurance generally has proven to be a lifesaver for unplanned mishappens and creating a system for insuring people against cancer could make a significant impact in lessening the cancer burden in Africa. Most of the existing health insurance schemes do not cover capital-intensive treatment like cancer and there is a need to revise this. Cancer is a leading cause of morbidity and mortality in Africa, it is ravaging our population and as such, we should attack it with similar force as it is attacking us. Stakeholders in health insurance need to revisit this issue to find a lasting solution. A lot of civil servants in Africa get charged monthly for pensions that they might not receive until many years after their service or never receive in their lifetime. Funds like this could better serve these individuals if they are contributed towards a health insurance scheme which could buffer the huge expenses of a disease like cancer.

#### Funding for cancer care and research

The usual predisposition for funding is to expect the government to provide all the funds. This is unrealistic in present-day Africa considering the economic instability and overwhelming challenges that already plague the continent. We need to begin to critically consider other funding sources.

Uncontrolled use of tobacco is a leading cancer risk factor. The use of tobacco products or second-hand smoking can increase cancer risk because chemical constituents of tobacco like acrylamide can damage the DNA and, deregulate cell division resulting in abnormal cell growth, ultimately cancer. Hence, tobacco-making companies contribute significantly to this environmental and public health issue. In this case, it is appropriate that such organizations should be important funders of cancer campaign programs, research, and treatment course to cushion the negative effect of their products. While smoking is a voluntary action, we cannot overlook the fact that some individuals are environmentally predisposed to tobacco as second-hand smokers and cannot directly control what is released into the environment.

As an example, the agenda for the generation of a national tobacco control policy in Nigeria dates to the 1950s, and a comprehensive Framework Convention for Tobacco Control (FCTC) complaint policy was developed in 2015 [61]. Poor funding and conflict of interest between protecting citizens from the harmful effect of tobacco and the economic reward from the industry are the significant barriers that have slowed the policy’s progress. The current “best buy” interventions in Nigeria include tax increases on tobacco products, smoke-free indoor workplaces and/or public places, bans on tobacco advertising, promotion and sponsorship, mass media campaigns on the possible danger of tobacco use and second-hand smoke and health information. While these are essential precautions, the policy should also cater to the already existing damages of tobacco and tobacco-related products by involving them as one of the major financiers of cancer education, care and research. Other industries whose products or by-products contribute indirectly to occupational or environmental carcinogens should also follow suit.

Not-for-profit and non-governmental organizations focused on cancer research and cancer care also should be well funded and supported to promote their effort and assist with their set objectives. It is also important to highlight the ongoing collaborative efforts in Africa to address cancer care inequities. Numerous research initiatives, screening programs, diagnostic advancements, and treatment interventions are being conducted across the region. These collaborative efforts involve multidisciplinary teams of healthcare professionals, researchers, and policymakers working together to improve cancer care outcomes. Furthermore, organizations such as the African Organization for Research and Training in Cancer (AORTIC) [62], the African Cancer Coalition (ACC), the African Palliative Care Association (APCA), and many more play a pivotal role in promoting cancer control and providing a platform for knowledge exchange and capacity building among healthcare professionals in Africa. This emphasizes the importance of funding these great initiatives and highlights the progress being made in combating cancer in sub-Saharan Africa. Africa is truly on a right path; however, a lot still needs to be done.

## Conclusion

Currently, the landscape of cancer care in Africa is appalling. The statistics are not encouraging and hence, there is a need for urgent interventions across Africa. Since everyone is at risk of cancer, we—Individuals stakeholders, institutions, and governmental and non-governmental organizations—need to join efforts together to achieve equitable access to cancer care in Africa.

Limitations to cancer care continue to reduce survival rates in Africa. Most of these limitations are interconnected, along with some other factors, contributing simultaneously to the growing burden of cancer in Africa. Therefore, addressing these limitations holistically is important to reduce the risk factors of populations currently at risk and improve the prospect of survival of cancer patients, and reduce the projection of cancer incidence and mortality by 2040.

## Supplementary Information

**Additional file 1.**

## Data Availability

Not applicable to this study.
