# Editorial Expression of Concern: Addressing cancer care inequities in sub‑Saharan Africa: current challenges and proposed solutions

**DOI:** 10.1186/s12939-024-02367-1

**Published:** 2025-01-09

**Authors:** Olabode Omotoso, John Oluwafemi Teibo, Festus Adebayo Atiba, Tolulope Oladimeji, Oluwatomiwa Kehinde Paimo, Farid S. Ataya, Gaber El‑Saber Batiha, Athanasios Alexiou

**Affiliations:** 1 Department of Biochemistry, University of Ibadan, Ibadan, Nigeria; 2 Department of Biochemistry and Immunology, Ribeirao Preto Medical School, University of Sao Paulo, Ribeirao Preto, Sao Paulo, Brazil; 3 Department of Zoology, University of Ibadan, Ibadan, Nigeria; 4 Department of Biochemistry, College of Biosciences, Federal University of Agriculture, Abeokuta, Nigeria; 5 Department of Biochemistry, College of Science, King Saud University, P.O. Box 2455, Riyadh, 11451 Saudi Arabia; 6 Department of Pharmacology and Therapeutics, Faculty of Veterinary Medicine, Damanhour University, AlBeheira, Damanhour, 22511 Egypt; 7 Department of Science and Engineering, Novel Global Community Educational Foundation, Hebersham, NSW 2770 Australia; 8 AFNP Med, Wien, 1030 Austria

**Editorial Expression of Concern to**: ***Int J Equity Health 22***,*** 189 (2023).***

**https://doi.org/10.1186/s12939-023-01962-y**

The Editors-in-Chief would like to inform the readers that following the publication of this article [1], concerns regarding incorrect statements, and discrepancies between statements in the published article & quoted references were raised (See below). During the investigation, the Publisher noted that the article has seen changes in authorship. The authors were unable to provide a satisfactory explanation of the raised concerns and documented proof of the contribution of added authors.

*1) In the statement ‘Low- and middle-income countries bear a disproportionate burden of the global cancer burden*,* accounting for approximately 400% and 168% respectively* [2] *’.*

*2) Genetic mutations or inherited gene mutations account for approximately 5–10% of all cancers*,* with some cancers having a stronger genetic component*,* such as breast and ovarian cancer in individuals with BRCA1 and BRCA2 mutations* [3].

*3) Gender also plays a role in cancer risk. Breast and cervical cancers are more prevalent in women*,* while prostate and testicular cancers are more frequent in men.*

*4)The statement ‘However*,* Africans have a relatively fair chance of cancer risk*,* with both incidence and mortality rates being comparatively lower than other continents’ is contradictory as compared to Table 1.*

The readers are therefore urged to take caution when interpreting the content of this article.

Author, Festus Adebayo Atiba did not respond to correspondence from the Publisher about this Editorial Express of Concern. All the remaining authors disagree with this Editorial Express of Concern.

## References

[1] Omotoso O, Teibo JO, Atiba FA, et al. Addressing cancer care inequities in sub-saharan Africa: current challenges and proposed solutions. Int J Equity Health. 2023;22:189. 10.1186/s12939-023-01962-y. 37697315 10.1186/s12939-023-01962-y PMC10496173

[6] Pramesh CS, Badwe RA, Bhoo-Pathy N, Booth CM, Chinnaswamy G, Dare AJ et al. Priorities for cancer research in low- and middle-income countries: a global perspective. Nat Med. 2022;28(4):649–57. Available from: https://www.nature.com/articles/s41591-022-01738-x. Cited 2022 Jun 25. 10.1038/s41591-022-01738-x PMC9108683 35440716

[7] Gbadegesin MA, Omotoso OE, Oluwasola TAO, Okolo CA, Soremekun O, Ogun GO, et al. Mutational analysis of p53 gene in cervical cancer and useful polymorphic variants in exons 3 and 4. Egypt J Med Hum Genet. 2021;22(23):1–8.38624675

